# Supplementary material for: Population exposure to multiple air pollutants and its compound episodes in Europe
Source: Nat Commun. 2024 Mar 13;15:2094. doi: 10.1038/s41467-024-46103-3 (PMC10937992; doi:10.1038/s41467-024-46103-3)
Supplement: Supplementary file 1 — Supplementary Information [file 41467_2024_46103_MOESM1_ESM.pdf]

Supplementary materials to "Population exposure to multiple air pollutants and its compound episodes in Europe"

Zhao-Yue Chen[1,2], Hervé Petetin[3], Raúl Fernando Méndez Turrubiates[1], Hicham Achebak[1,4], Carlos Pérez García-Pando[3,5] and Joan Ballester[1]

1 ISGLOBAL, Barcelona, Spain
2 Universitat Pompeu Fabra (UPF), Barcelona, Spain
3 Barcelona Supercomputing Center, Barcelona, Spain
4 Inserm, France Cohortes, Paris, France
5 ICREA, Catalan Institution for Research and Advanced Studies, Barcelona, Spain

Correspondence: Zhao-Yue Chen (zhaoyue.chen@isglobal.org)

Contents

1 Supplemental methods description **3**

1.1 Data source **3**

Table S1. The list of variables used in this study **4**

1.2 Selected Variable in models **6**

Table S2. The list of variables selected for four air pollutant models **7**

1.3 Distance-weighted loss function and validation metrics **9**

1.4 Formula of calculating the indicators **9**

1.4.1 Threshold of WHO Guidance: 9

Table S3. Summaries of WHO air quality guidelines **9**

1.4.2 Concentration Indicators: 9

1.4.3 Cumulative time of exposure Indicators: 10

1.4.4 Population Indicators: 10

2 Other Figures **12**

Figure S1. The average observed (left) and model-estimated (right) PM2.5, PM10, NO2, MDA8(maximum daily 8h average) O3 concentrations from 2003 to 2019 (8 Maps) 12

Figure s2. Top 20 Most Important Features (ordered by model gains) and Their Percentage Contribution to PM2.5, PM10, NO2, and O3 Models 20

Figure S3. Spatial cross-validation results for PM2.5 (upper panel), PM10 (lower panel) models 13

Figure s4. The composition of compound days exceeding WHO Daily Limits for multiple pollutants in different months 16

Figure s5. The overall composition of unclean-air exposure time (including compound days) between 2003-19 for PM25, PM10 , NO2 and Ozone 15

Figure s6. Time evolution in the composition of unclean-air exposure time (including compound days) for PM25 and PM10 17

Figure s7. Time evolution in the population(%) in long-term clean-air areas for each pollutants in different regions.......... 19

3 Other Tables 21

Table S4. Descriptive statistics of observed and correpsonding QML grid-estimated PM2.5, PM10, NO2, MDA8(maximum daily 8h average) O3 concentrations in different regions (South, East, West, and North) of Europe. 21

Table S5. Comparison of Spatial Out-of-Sample Predicted QML Products, CAMSRA, and MERRA-2 Products with Ground Level Observations in Different Regions of Europe. 21

Table S6. Annual Performance of Temporal Cross-Validation in QML Products 23

Table s7. Comparison in European population proportion suffering from long-term unclean-air (exceeding WHO annual or peak season guideline) for PM2.5, PM10, NO2 and Ozone among different studies.......... 28

Table s8. European population proportion (%) exposed to at least 1 day per year of multi-pollutant compound unclean-air days and four major combinations. 26

Table s9. Average unclean-air exposure time (unit: days) per year for multi-pollutant compound unclean-air days and four major combinations. 27

Reference 29

1 Supplemental methods description

1.1 Data source

The daily mean observations of PM2.5, PM10, NO2 and O3_8h were collected from two main databases in European environment information and observation network (Eionet): the Airbase (2003-2012) and the Air Quality e-Reporting (2013-2019). Figure S1 illustrates the concentration distribution of background monitoring stations across the 36 European countries. Specifically, there are approximately 1310 stations for PM2.5, 3438 for PM10, 1867 for NO2, and 2021 for O3.

For the aerosol product, we used the total aerosol optical depth (AOD), Fine-mode AOD (fAOD), and Coarse-mode AOD (cAOD) products generated from our previous works[1]. They are based on models with AERONET (AErosol RObotic NETwork) observations, Gap-filled satellite AOD, climate and atmospheric composition reanalyses. These products formed a daily AOD dataset with a resolution of 0.1 degrees, covering the entire European region. These products exhibit better quality with an out-of-sample R2 equal to 0.68 for AOD, 0.66 for fAOD and 0.65 for cAOD, compared with corresponding satellite or reanalysis products, respectively. Over 91.6%, 81.6%, and 88.9% of QML AOD, fAOD and cAOD predictions fall within ±20% Expected Error (EE) envelopes, respectively.

Reanalysis meteorological data primarily came from the ERA5 land dataset, which is the fifth-generation European Centre for Medium-Range Weather Forecasts reanalysis dataset. It provided various variables, including boundary layer height, ambient temperature, ambient dew point temperature, wind speed at 10 m height above sea level, surface pressure, surface solar radiation, total precipitation, and total evaporation, at a spatial resolution of 0.1° × 0.1°.

Air quality reanalysis data were collected from the CAMSRA (Copernicus Atmosphere Monitoring Service Reanalysis) dataset, with a resolution of 0.75° × 0.75°, and from the MERRA-2 (Modern-Era Retrospective analysis for Research and Applications, version 2) dataset, with a resolution of 0.625° × 0.5°, to obtain atmospheric composition information.

Land use information was obtained from various sources. Road density data [2] for different road types (Highways, Primary roads, Secondary roads, Tertiary roads, and Local roads) were obtained from the Global Roads Inventory Project (GRIP) dataset at a resolution of approximately 5 arcminutes (~8x8 km). Leaf area index and other land-related variables were collected from the ERA5_land dataset. Local climate zone [3] data were sourced from the Urban Database and Access Portal Tools (WUDAPT) at a resolution of 1 km. Emission data, encompassing natural and anthropogenic sources were included from the CAMSRA global emission inventories.

To calculate population-weighted indicators, high-resolution gridded population data from the Gridded Population of the World, Version 4 (GPWv4) database were obtained at a spatial resolution of 30 arc-seconds (~1 km2) for the years 2000, 2005, 2010, 2015, and 2020. Linear regression was used to interpolate population data for the remaining years between 2003 and 2019[4]. The gridded population data were upscaled to a 0.1-degree grid cell using the area-weighted sum method: the values of 1 km grid cells were aggregated based on their relative areas within the 0.1-degree grid cell.

Table S1. The list of variables used in this study

SHORT NAME	SOURCE	RESOLUTION	LONG NAME	UNIT
**PM2.5, PM10, NO2, O3 MDA8**	European environment information and observation network (Eionet)	government air quality monitoring station data	Observed station Based PM2.5, PM10, NO2 and O3 MDA8	μg/m3
**ERA5L_U10**	ERA5_land	hourly data from 2003-2019, 0.1° × 0.1°	10m u component of wind	m s**-1
**ERA5L_V10**			10m v component of wind	m s**-1
**LAI_HV**			leaf area index high vegetation	m**2 m**-2
**LAI_LV**			leaf area index low vegetation	m**2 m**-2
**MSDWSWRF**			Surface solar radiation downwards	J m**-2
**ERA5L_ASN**			snow albedo	(0 - 1)
**ERA5L_SP**			surface pressure	Pa
**ERA5L_ST1**			Soil surface temperature	(0 - 1)
**ERA5L_SLHF**			Surface latent heat flux	J m**-2
**ERA5L_TE**			total evaporation	m
**ERA5L_D2M**			Daily mean dewpoint temperature	K
**ERA5L_T2M**			Daily mean temperature	K
**EOBS_PP**	E-OBS	Daily data from 2003-2019, 0.1° × 0.1°	Precipitation amount	mm
**EOBS_HU**			Relative humidity	%
**EOBS_RR**			Surface shortwave downwelling radiation	W/m2
**EOBS_TG**			Maximum temperature	°C
**EOBS_TN**			Mean temperature	°C
**EOBS_TX**			Minimum temperature	°C
**TAODPRE**	Previous studies	Daily data from 2003-2019, 0.1° × 0.1°	Total AOD	\
**FAODPRE**			Fine mode AOD	\
**BLD**	ERA5	hourly data from 2003-2019, 0.25° × 0.25°	boundary layer dissipation	J m**-2
**BLH**			boundary layer height	m
**HCC**			high cloud cover	(0 - 1)
**TCC**			total cloud cover	(0 - 1)
**LCC**			low cloud cover	(0 - 1)

SHORT NAME	SOURCE	RESOLUTION	LONG NAME	UNIT
**TCO3**			total column ozone	J m**-2
**TP**			total precipitation	m
**ALUVD**			uv visible albedo for diffuse radiation	(0 - 1)
**ALUVP**			uv visible albedo for direct radiation	(0 - 1)
**POP**	Gridded Population of the World (GPW), v4	years from 2000, 2005, 2010, 2015, and 2020, 30 arcs second	Population	\
**CAMS_SSAOD550**	CAMSRA	Three hourly data from 2003-2019, 0.75° x 0.75°	sea salt aerosol optical depth 550nm	\
**CAMS_CO**			Carbon monoxide	kg m-2
**CAMS_GO3**			Ozone	kg m-2
**CAMS_PM10**			Particulate matter d < 10 µm (PM10)	kg m-2
**CAMS_SO2**			Sulphur dioxide	kg m-2
**CAMS_PM2P5**			Particulate matter d < 2.5 µm (PM2.5)	kg m-2
**CAMS_PM1**			Particulate matter d < 1 µm (PM1)	kg m-2
**MERRA_SSSMASS25**	MERRA-2	hourly data from 2003-2019,0.625°×0.5°	Sea Salt Surface Mass Concentration - PM 2.5	kg m-3
**MERRA_BCSMASS**			Black Carbon Surface Mass Concentration	kg m-3
**MERRA_SO4SMASS**			SO4 Surface Mass Concentration	kg m-3
**MERRA_DUSMASS**			Dust Surface Mass Concentration	kg m-3
**MERRA_SO2SMASS**			SO2 Surface Mass Concentration	kg m-3
**SO2_RES**	CAMS global emission inventories	monthly data from 2003-2019,0.1°x0.1°,	Anthropogenic emissions of so2 from residential	kg m-2 s-1
**OC_SUM**			Anthropogenic emissions of organic carbon	kg m-2 s-1
**NMVOC_IND**			Anthropogenic emissions of non-methane VOCs (volatile organic chemicals) from industry	kg m-2 s-1

SHORT NAME	SOURCE	RESOLUTION	LONG NAME	UNIT
OTHERVOCS_IND			Anthropogenic emissions of other VOCs from industry	kg m-2 s-1-
SO2_IND			Anthropogenic emissions of so2 from industry	kg m-2 s-1-
NOX_RES			Anthropogenic emissions of nitrogen oxides from residential	kg m-2 s-1
NMVOC_SLV			Anthropogenic emissions of non-methane VOCs (volatile organic chemicals) from Solvents production	kg m-2 s-1
NH3_TRO			Anthropogenic emissions of ammonia from road transport	kg m-2 s-1
NOX_TRO			Anthropogenic emissions of nitrogen oxides from road transport	kg m-2 s-1
NOX_SUM			Anthropogenic emissions of nitrogen oxides	kg m-2 s-1
OTHERVOCS_SUM			Anthropogenic emissions of other VOCs	kg m-2 s-1
GRIP4_TOAL_DENS_M_KM2	GRIP global roads database	Fixed, 5 arcminutes resolution (~8x8km).	Total road density, all types combined	\
GRIP4_TP1_DENS_M_KM2			Type 1 density (highways)	\
GRIP4_TP2_DENS_M_KM2			Type 2 density (primary roads)	\
GRIP4_TP3_DENS_M_KM2			Type 3 density (secondary roads)	\
GRIP4_TP4_DENS_M_KM2			Type 4 density (tertiary roads)	\
GRIP4_TP5_DENS_M_KM2			Type 5 density (local roads)	\
LCZ	world urban dataset	1km	Local climate Zone	\
CLIMATE	KÖPPEN-GEIGER CLIMATE CLASSIFICATION	5 arc min	Climate Classification	\

1.2 Selected Variable in models

Under the Boruta feature selection procedure, the selected variables for each air pollutant model are listed in Table S2.

Table S2. The list of variables selected for four air pollutant models

SHORT NAME	LONG NAME	MODEL			
		PM2.5	PM10	NO2	O3
**ERA5L_U10**	10m u component of wind	√	√	√	√
**ERA5L_V10**	10m v component of wind	√	√	√	√
**LAI_HV**	leaf area index high vegetation	√	√	√	√
**LAI_LV**	leaf area index low vegetation	√	√		
**ERA5L_SP**	surface pressure	√		√	
**ERA5L_STL1**	Soil surface temperature	√		√	√
**ERA5L_SLHF**	Surface latent heat flux		√	√	√
**ERA5L_TE**	total evaporation		√		√
**ERA5L_TP**	total precipitation	√	√	√	
**ERA5L_SKT**	Skin temperature		√		
**ERA5L_SSR**	Surface net solar radiation		√		√
**ERA5L_D2M**	Daily mean dewpoint temperature			√	√
**ERA5L_T2M**	Daily mean temperature	√	√	√	
**EOBS_PP**	Precipitation amount	√	√	√	√
**EOBS_HU**	Relative humidity	√	√	√	√
**EOBS_RR**	Surface shortwave downwelling radiation	√	√	√	
**EOBS_TG**	Minimum temperature	√	√		
**EOBS_TN**	Mean temperature	√	√	√	
**EOBS_TX**	Maximum temperature	√	√	√	√
**TAODPRE**	Total AOD	√	√	√	
**FAODPRE**	Fine mode AOD	√		√	√
**BLH**	boundary layer height	√	√		
**MCC**	medium cloud cover			√	
**TCC**	total cloud cover			√	√
**LCC**	low cloud cover		√	√	
**TCO3**	total column ozone		√	√	√
**TSR**	Top net solar radiation	√	√		√
**ALUVD**	uv visible albedo for diffuse radiation	√	√		
**ALUVP**	uv visible albedo for direct radiation		√		
**CAMS_SSAOD550**	sea salt aerosol optical depth 550nm	√	√	√	√
**CAMS_OMAOD550**	organic matter aerosol optical depth 550nm		√	√	
**CAMS_DUAOD550**	dust aerosol optical depth 550nm			√	
**CAMS_CO**	Carbon monoxide	√	√	√	√
**CAMS_GO3**	Ozone	√	√	√	√
**CAMS_PM10**	Particulate matter d < 10 μm (PM10)	√	√		
**CAMS_SO2**	Sulphur dioxide	√	√	√	
**CAMS_NO2**	nitrogen dioxides	√		√	√
**CAMS_NO**	nitrogen oxides		√	√	
**CAMS_PM2P5**	Particulate matter d < 2.5 μm (PM2.5)	√	√	√	

SHORT NAME	LONG NAME	MODEL			
		PM2.5	PM10	NO2	O3
CAMS_PM1	Particulate matter d < 1 μm (PM1)	√			
MERRA_SSSMASS25	Sea Salt Surface Mass Concentration - PM 2.5	√	√	√	√
MERRA_BCSMASS	Black Carbon Surface Mass Concentration	√	√	√	√
MERRA_SO4SMASS	SO4 Surface Mass Concentration	√	√	√	√
MERRA_DUSMASS	Dust Surface Mass Concentration	√	√	√	√
MERRA_SO2SMASS	SO2 Surface Mass Concentration	√	√		
SO2_RES	Anthropogenic emissions of so2 from residential		√	√	√
CO_RES	Anthropogenic emissions of carbon monoxide from residential		√		
CO_SUM	Anthropogenic emissions of carbon monoxide		√		
OC_SUM	Anthropogenic emissions of organic carbon		√	√	√
NMVOC_IND	Anthropogenic emissions of non-methane VOCs (volatile organic chemicals) from industry	√		√	√
OTHERVOCS_IND	Anthropogenic emissions of other VOCs from industry	√			
OTHERVOCS_TRO	Anthropogenic emissions of other VOCs from road transport		√		
SO2_IND	Anthropogenic emissions of so2 from industry	√		√	
NOX_RES	Anthropogenic emissions of nitrogen oxides from residential	√		√	√
NH3_TRO	Anthropogenic emissions of ammonia from road transport	√			
NOX_TRO	Anthropogenic emissions of nitrogen oxides from road transport	√		√	√
NMVOC_SLV	Anthropogenic emissions of non-methane VOCs (volatile organic chemicals) from Solvents production	√		√	
NMVOC_SUM	Anthropogenic emissions of non-methane VOCs (volatile organic chemicals)	√	√		
NOX_SUM	Anthropogenic emissions of nitrogen oxides	√			
OTHERVOCS_SUM	Anthropogenic emissions of other VOCs	√	√	√	√
GRIP4_TOAL_DENS_M_KM2	Total road density, all types combined	√		√	
GRIP4_TP1_DENS_M_KM2	Type 1 density (highways)	√	√	√	√
GRIP4_TP2_DENS_M_KM2	Type 2 density (primary roads)	√	√	√	√
GRIP4_TP3_DENS_M_KM2	Type 3 density (secondary roads)	√	√	√	√
GRIP4_TP4_DENS_M_KM2	Type 4 density (tertiary roads)		√	√	√
GRIP4_TP5_DENS_M_KM2	Type 5 density (local roads)		√		√
LCZ	Local climate Zone	√	√	√	√
CLIMATE	Climate Classification	√	√	√	√

1.3 Distance-weighted loss function and validation metrics

Distance-weighted loss function:

The mathematical formulation for distance-weighted loss function represented as:

$$W_i = \frac{(D_i - D_{min})}{(\bar{D} - D_{min})}$$

$$L(Y, Y^*) = \sum_{i=1}^{n} W_i * L(y_i, y_i^*)$$

where D_i is the distance of station *i* to its nearest site; D_{min} and $\bar{D}$ denote the minimum and average of D_i in Europe; and $L(Y, Y^*)$ is the overall loss function and $L(y_i, y_i^*)$ the loss functions for station *i* (y_i and y_i^* are the observations and predictions at station *i*).

Validation metrics:

$$\text{Normalized Mean Bias (NMB \%)} = \frac{\sum_{k=1}^{n}(Pred_k - Obs_k)}{\sum_{k=1}^{n}(Obs_k)}$$

$$\text{Normalized Root Mean Square Error (NRMSE \%)} = \frac{\sqrt{\frac{\sum_{k=1}^{n}(Pred_k - Ob\ _k)^2}{N}}}{IQR(Obs)}$$

Where *Pred* is the model prediction; *Obs* is the observation and *n* is the total numbers of observations.

1.4 Formula of calculating the indicators

1.4.1 Threshold of WHO Guidance:

Table S3. Summaries of WHO air quality guidelines

Pollutants	Short-term exposure Guideline		Long-term exposure Guideline	
	**24h**	**8h maximum**	**Annual**	**Peak Season**
**PM2.5**	15	\	5	\
**PM10**	45	\	15	\
**NO2**	25	\	10	\
**O3**	\	100	\	60 (Interim Target 2: 70)
**Clean-air Areas**	Less than 4 exceedance days Per Year (99th percentile of daily values within guidelines)		Long-term concentrations within corresponding guideline	

1.4.2 Concentration Indicators:

By using the gridded model estimations, we calculated the daily region population-weighted (PW) average concentrations for each NUTs 3 units, countries levels or continent levels. The daily region PW concentration for a given area was identified as:

$$PW\ concentration_R = \frac{\sum_g^R (Pop_g \times C_g)}{\sum_g^R Pop_g}$$

Where $PW\ concentration_R$ represents the pop-weighted concentration on area *R*; Pop_g is the GPWv4 population of grid g in the corresponding year; C_g stands for the grid concentration on grid *g*; $\sum_g^R Pop_g$ is the total Population of area *R*, and Rn here is the all grid within area *R*.

1.4.3 Cumulative time of exposure Indicators:

We calculated annual unclean-air exposure time for single pollutant exceeding the daily limit (Table S3), or multiple pollutants joint exceeding daily limits (or Compound days).

The calculation of annual unclean-air exposure time for single pollutant has two steps: first is to obtain the annual unclean-air person-days for each gird, second is to obtain the annual regional PW average unclean-air days.

The annual unclean-air person-days for each gird cell was identified as:

$$Annual\ unclean - air\ person - days\ _{gY} = \sum_{d}^{Y} Pop_g \times I_{gd}(C_{gd} > limit)$$

Where annual unclean − air person − days $_{gY}$ is the cumulative unclean-air person-days in grid g for specified year Y; Pop_g represents the population of grid g in the corresponding year; $I_{gd}(C_{gd} > limit)$ is the Boolean value indicating whether concentration C_{gd} on grid g and day d exceeds WHO daily limit.

The annual regional PW average unclean-air days was identified as:

$$PW\ unclean\text{-}air\ days = \frac{\sum_g^R \text{annual unclean} - \text{air person} - \text{days}_{gY}}{\sum_g^R Pop_g}$$

Where *PW unclean-air days* is the pop-weighted unclean-air day on area R and year Y; $\sum_g^R$ annual unclean − air person − days$_{gY}$ is the sum of annual unclean-air person-days for all grids g within area R for specified year Y; $\sum_g^R Pop_g$ is the total Population of area R.

The calculation of annual unclean-air exposure time for multiple pollutant (or Compound days) is similar to the single pollutant's indicator, by replacing the single pollutant' exceedances with multiple pollutants' exceedances.

The annual Compound person-days is as follows:

$$Annual\ compound\ person - days\ _{gY} = \sum_{d}^{Y} Pop_g \times \prod_{p} I_{pgd}(C_{pgd} > limit_p)$$

Where *annual compound person − days* $_{gY}$ is the cumulative compound person-days in grid g for specified year Y; Pop_g is the population of grid g in the corresponding year; $I_{pgd}(C_{pgd} > limit_p)$ is the Boolean value for whether concentration of pollutant C_{pgd} on grid g and day d exceed its WHO daily limit; $\prod_p I_{pgd}(C_{pgd} > limit_p)$ is the multiplication of Boolean values for each pollutant, represented the Boolean value for compound events.

The annual regional PW Compound unclean-air days is as follows:

$$PW\ compound\ unclean\text{-}air\ days = \frac{\sum_g^R \text{annual compound person}-\text{days}_{gY}}{\sum_g^R Pop_g}$$

Where *PW compound unclean-air days* is the PW compound unclean-air day on area R and year Y; $\sum_g^R$ annual compound person − days$_{gY}$ is the sum of annual compound person-days for all grids g within area R for specified year Y; $\sum_g^R Pop_g$ is the total Population of area R.

1.4.4 Population Indicators:

We mainly calculate the proportion of individuals residing in short-term and long-term

clean-air areas (threshold listed in Table S3), respectively.

The annual proportion in short-term clean-air areas is identified as follows:

$$proportion\ in\ short - term\ safe\ air\ areas = \frac{\sum_g^R (Pop_g \times I_{gy}(Unsafe\ day_{gy} > limit))}{\sum_g^R Pop_g}$$

Where $proportion\ in\ short - term\ safe\ air\ areas$ is the proportion of people living in area R where 99% of days in year y comply with WHO daily limits; $Unsafe\ day_{gy}$ represents the counts of days over daily limits in grid g and year y; $I_{gy}(Unsafe\ day_{gy} > limit)$ is the Boolean value for whether the numbers of $Unsafe\ day_{gy}$ on grid g exceed the limit (<1% unclean-air days for year y, around 3-4 days); $\sum_g^R (Pop_g \times I_{gy}(Unsafe\ day_{gy} > limit))$ is the population living in area R where 99% of days in year y comply with WHO daily limits

The annual proportion in long-term clean-air areas is identified as follows:

$$Proportion\ in\ long - term\ safe\ air\ areas = \frac{\sum_g^R (Pop_g \times I_{gy}(C_{gy} \leq limit))}{\sum_g^R Pop_g}$$

Where $proportion\ in\ long - term\ safe\ air\ areas$ is the proportion of people living in area R where meet the WHO annual or peak-season guideline; $I_{gy}(C_{gy} \leq limit)$ is the Boolean value for whether the whether annual or peak-season concentration C_{gy} on grid g and year y exceed WHO long-term guideline; $\sum_g^R (Pop_g \times I_{gy}(C_{gy} \leq limit))$ is the population living in area R where meet the WHO annual or peak-season guideline.

2 Other Figures

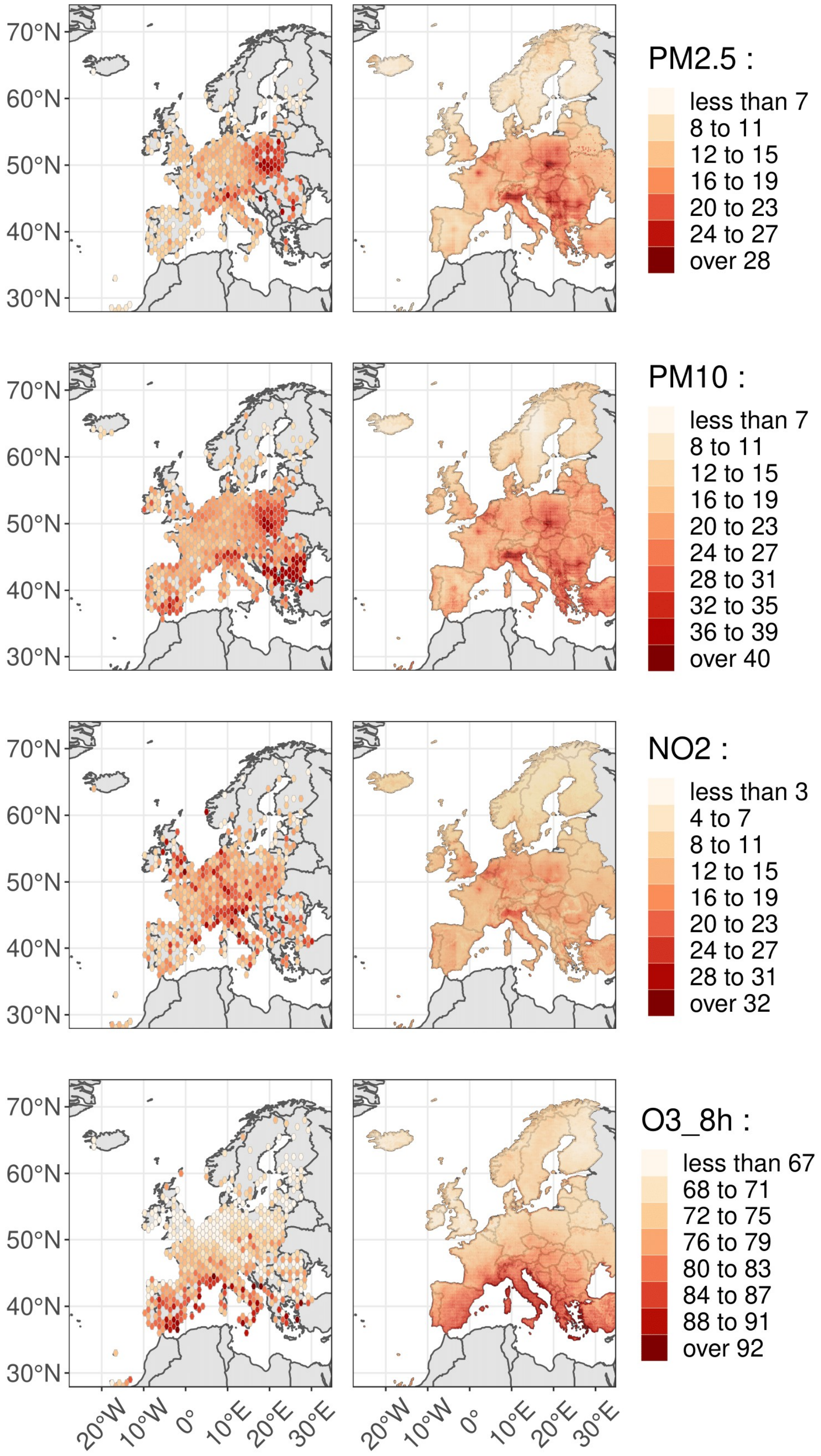

Figure S1. The average observed (left) and model-estimated (right) PM2.5, PM10, NO2, MDA8(maximum daily 8h average) O3 concentrations from 2003 to 2019 (8 Maps)

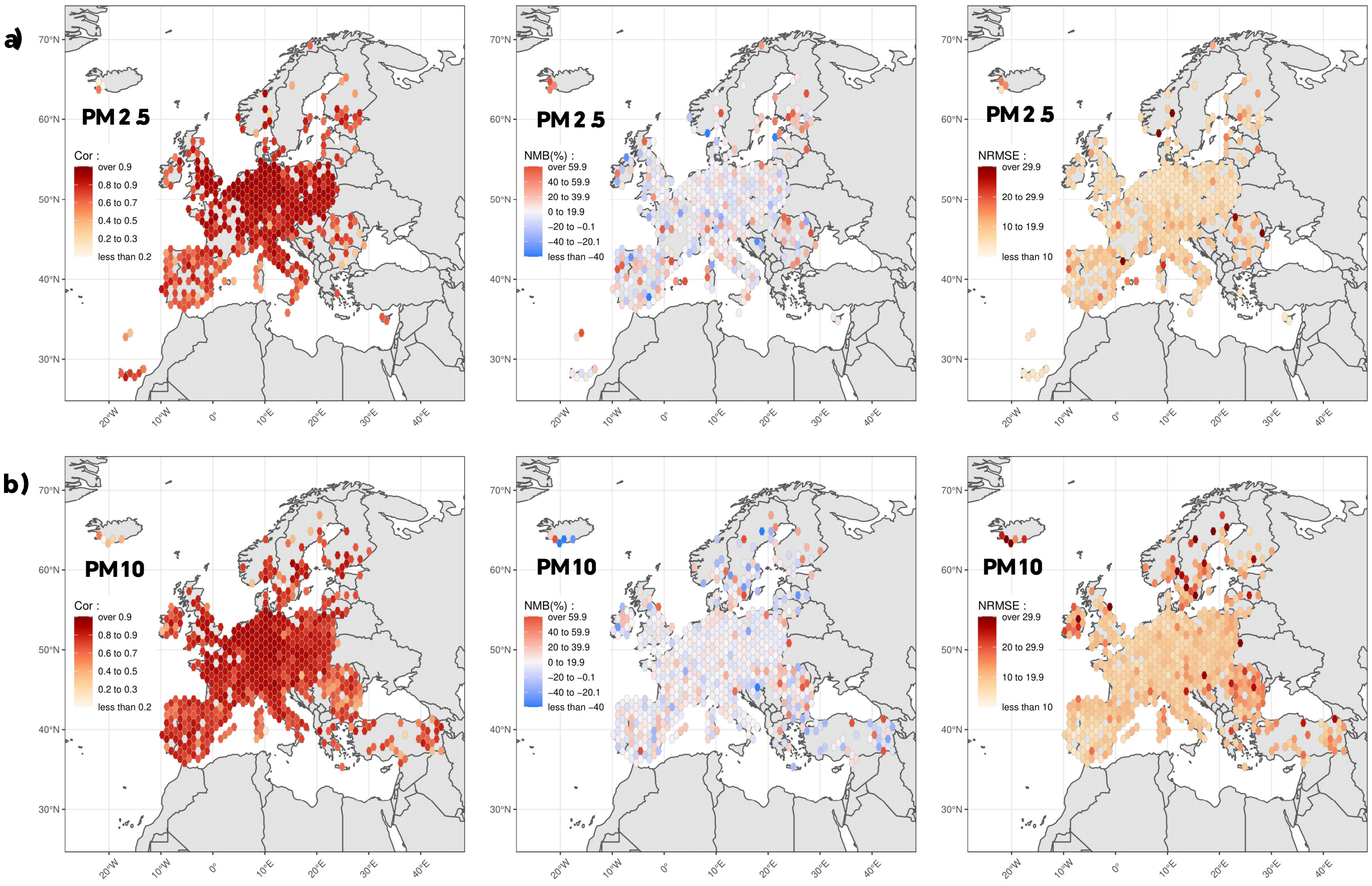

Figure S2. Spatial cross-validation results for PM2.5 (a), PM10 (b) models

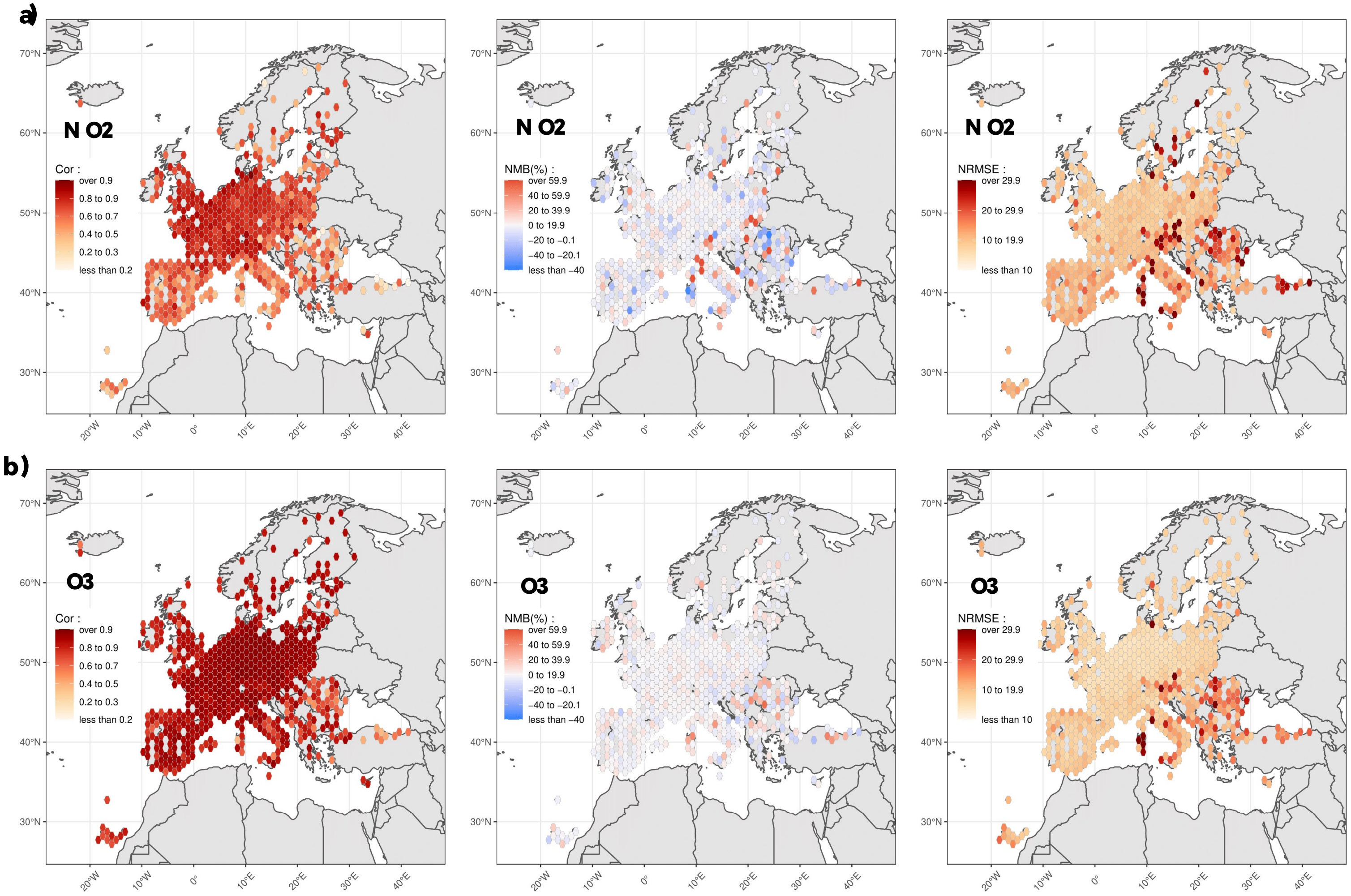

Figure S3. Spatial cross-validation results for NO2 (a), MDA8(maximum daily 8h average) O3 (b) models

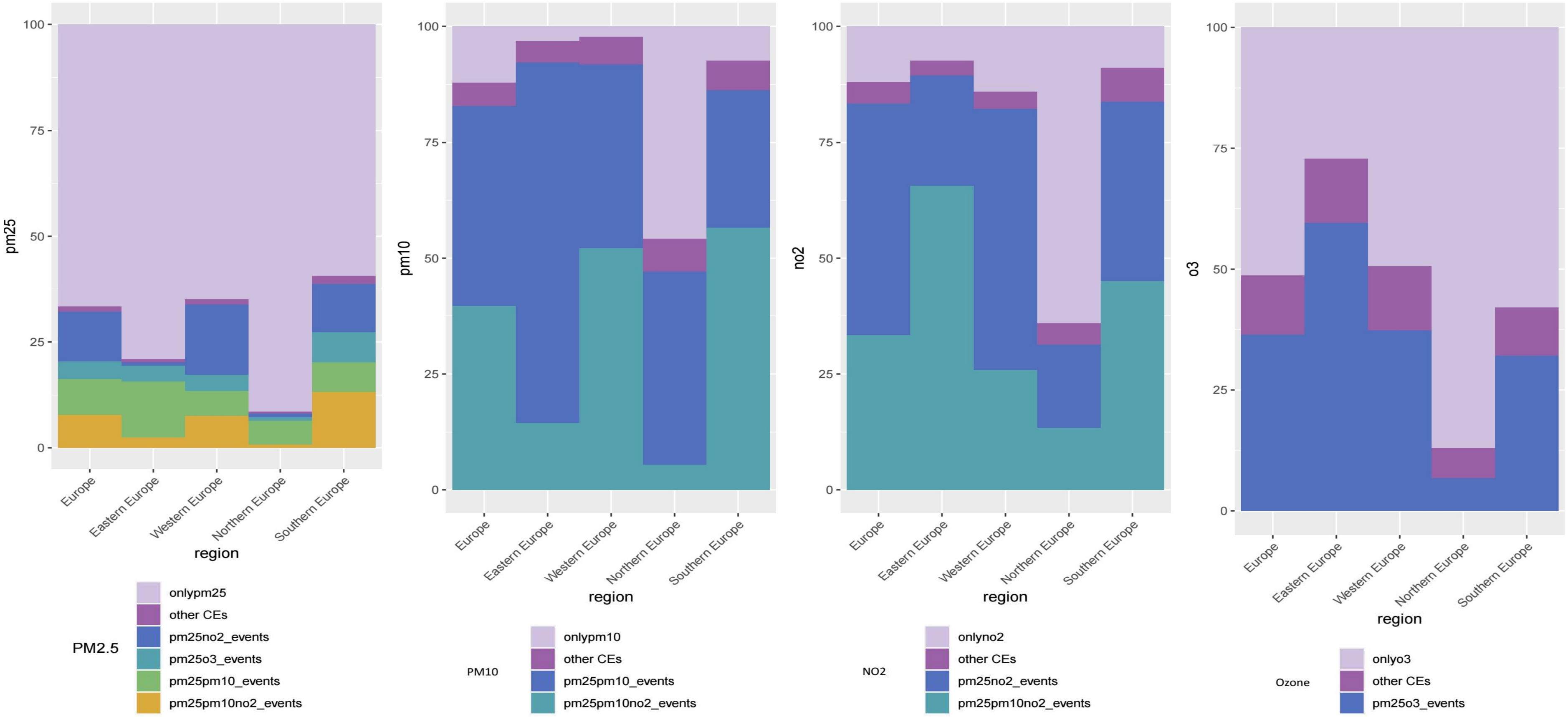

Figure s4. The overall composition of unclean-air exposure time (including compound days) between 2003-19 for PM25, PM10 , NO2 and Ozone

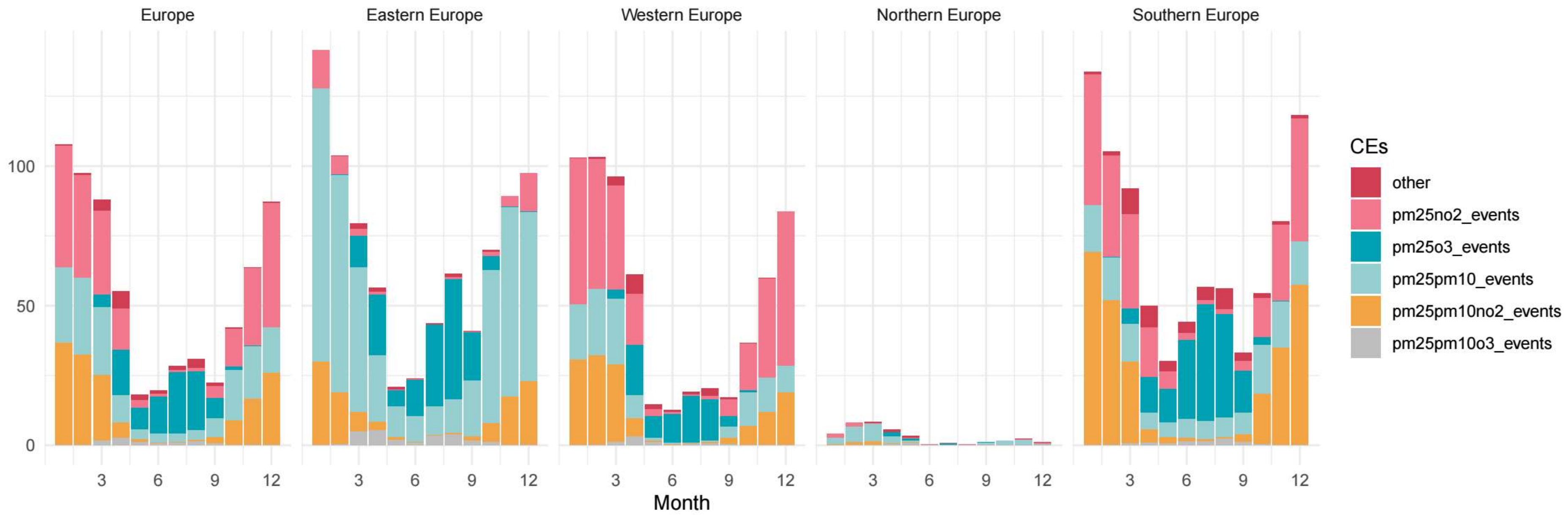

Figure s5. The composition of Compound Days exceeding WHO Daily Limits for multiple pollutants in different months

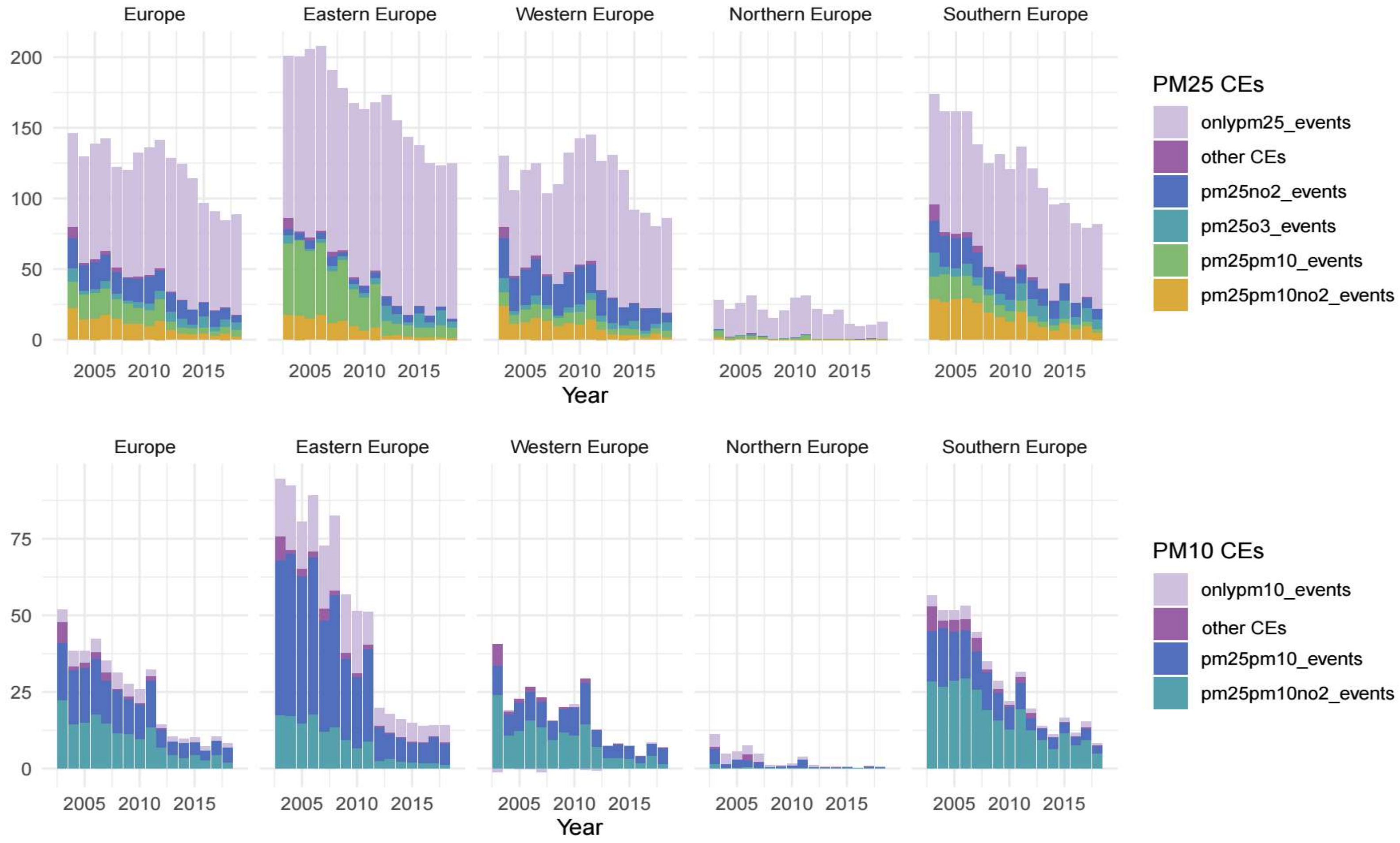

Figure s6. Time evolution in the composition of unclean-air exposure time (including Compound days) for PM25 and PM10

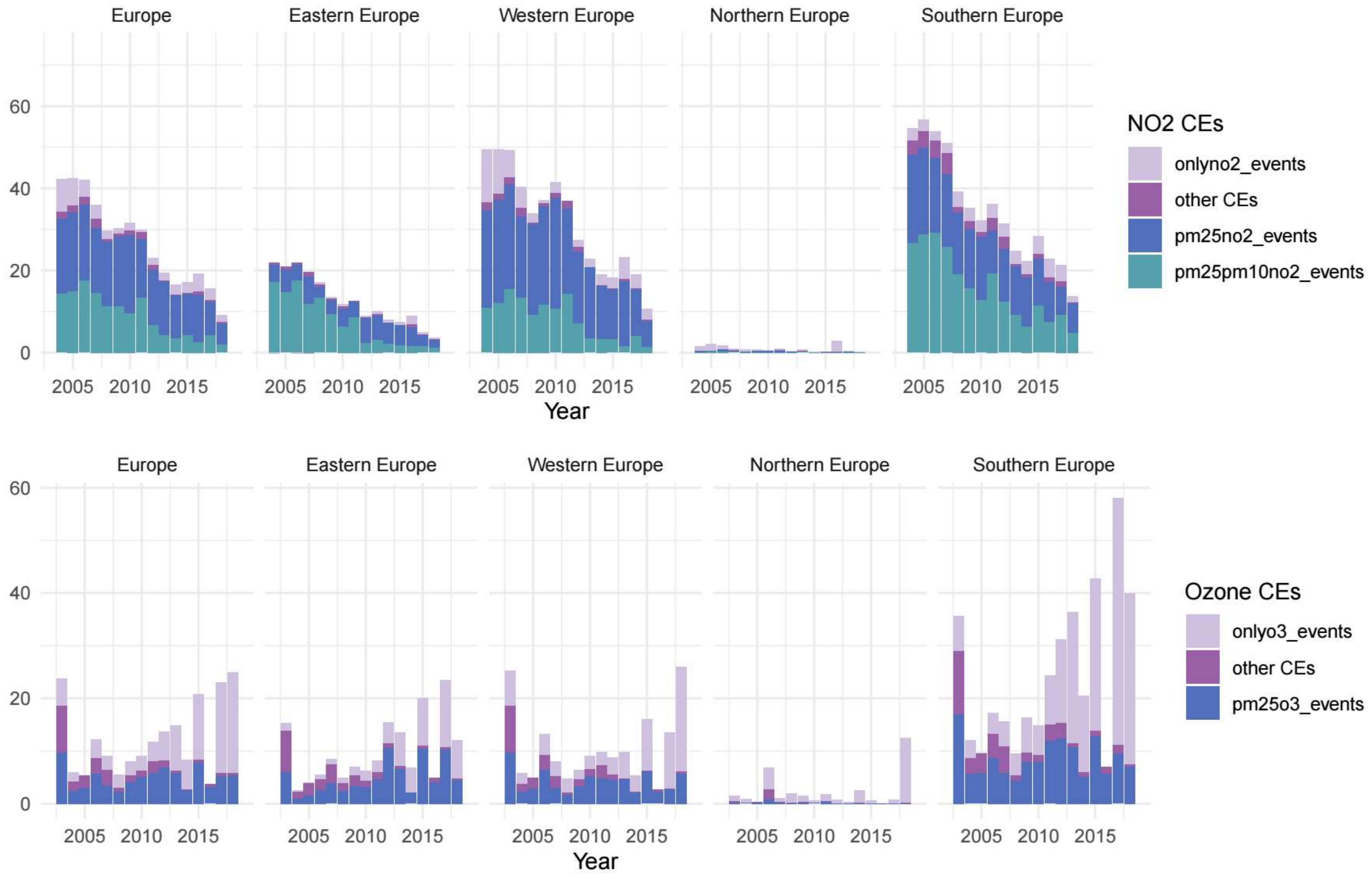

Figure s7. Time evolution in the composition of unclean-air exposure time (including Compound days) for NO2 and Ozone

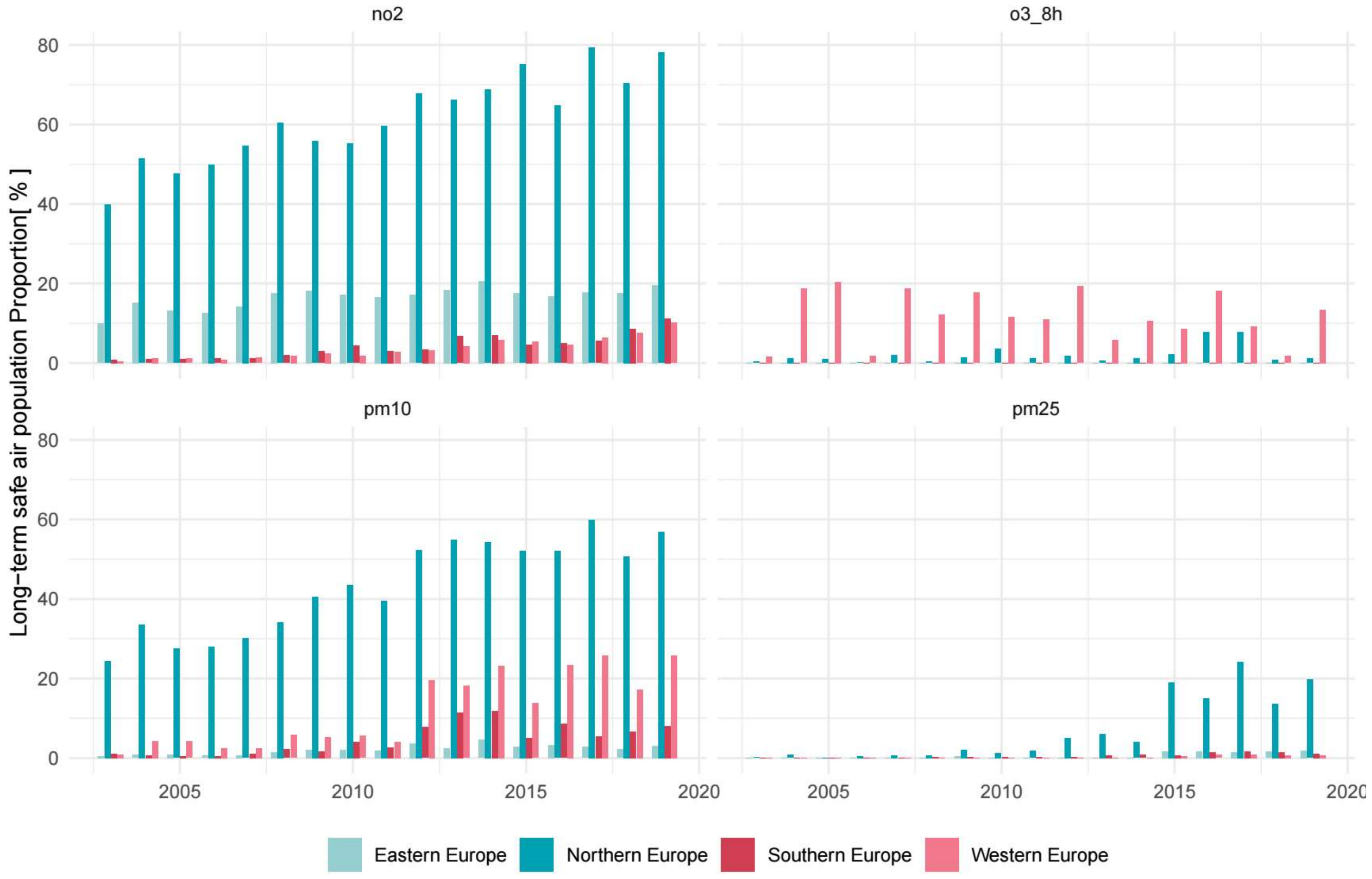

Figure s7. Time evolution in the population(%) in long-term clean-air areas for each pollutants in different regions

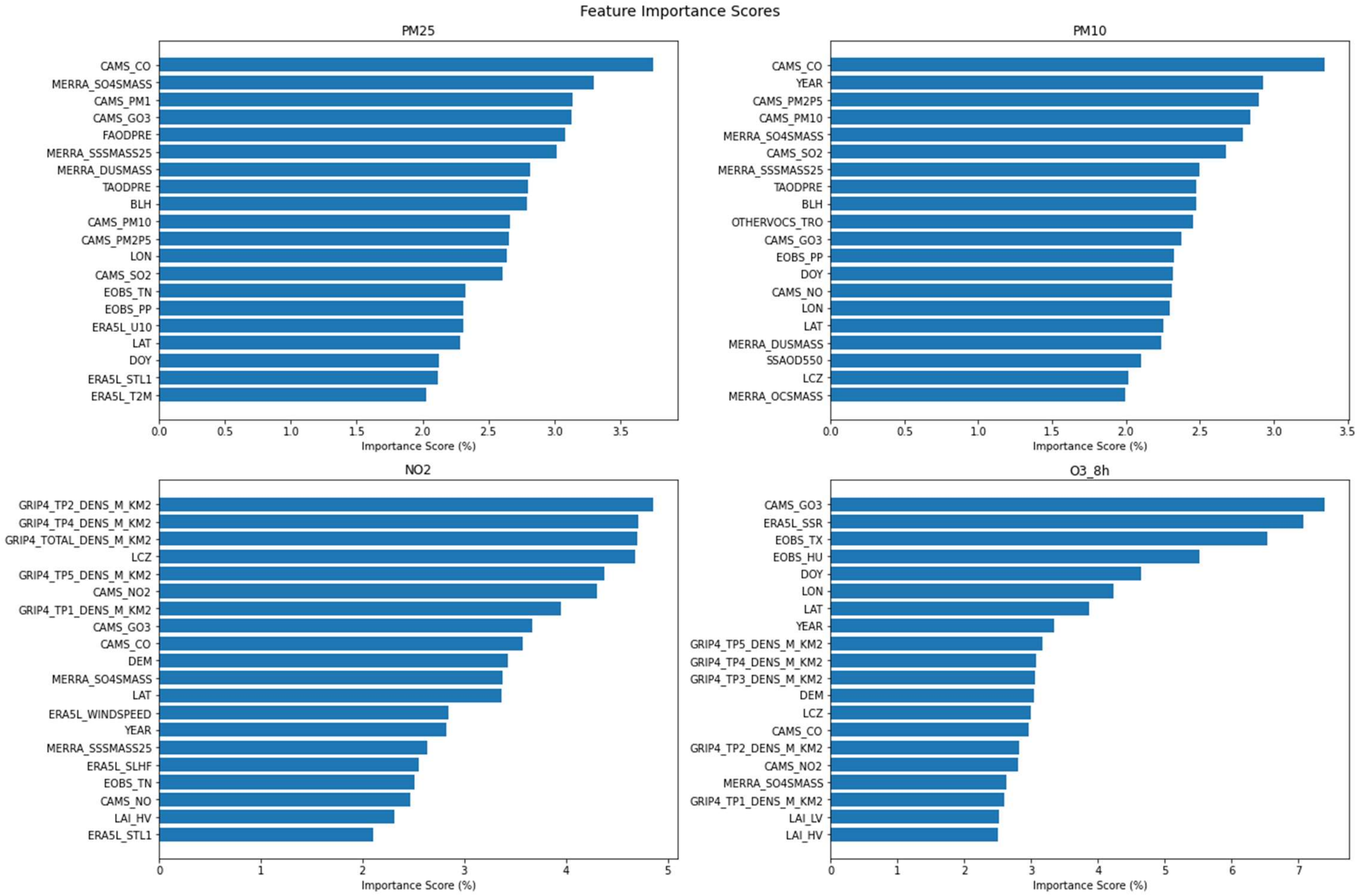

Figure s8. Top 20 Most Important Features (ordered by model gains) and Their Percentage Contribution to PM2.5, PM10, NO2, and MDA8 (maximum daily 8h average) O3 Models

3 Other Tables

Table S4. Comparison of Spatial Out-of-Sample Predicted QML Products, CAMSRA, and MERRA-2 Products with Ground Level Observations in Different Regions of Europe.

Note: as CAMSRA do not have MDA8(maximum daily 8h average) O3 observation, we used CAMSRA O3 to compare with ground -level O3 instead. Due to data limitations, the comparison of MERRA-2 is not performed for NO2 and O3.

				QML			CAMSRA			MERRA-2		
**Variable**	region	Nsite	Nobs	Cor	NMB (%)	NRMSE (%)	Cor	NMB (%)	NRMSE (%)	Cor	NMB (%)	NRMSE (%)
**PM25**	Europe	1,310	3,435,307	0.8	-0.94	1.84	0.48	-1.17	2.82	0.54	3.16	2.73
	Western Europe	616	1,379,372	0.83	-0.58	2.02	0.47	-2.34	2.94	0.57	6.26	2.78
	Southern Europe	500	1,442,244	0.81	0.64	1.58	0.49	4.79	2.68	0.52	4.59	2.67
	Eastern Europe	85	192,279	0.75	0.02	2.69	0.36	-23.44	3.71	0.52	-20.53	3.54
	Northern Europe	109	421,412	0.72	1.86	1.47	0.4	-9.1	1.83	0.41	-9.37	1.77
**PM10**	Europe	2,438	3,226,925	0.79	-3.81	2.71	0.45	-15.49	3.68	0.32	-17.57	4.11
	Western Europe	1,146	1,467,153	0.8	-1.93	2.06	0.5	-5.76	3.28	0.34	-22.75	3.88
	Southern Europe	849	1,297,056	0.78	-2.77	1.97	0.42	-20.25	3.76	0.27	-9.7	4.09
	Eastern Europe	298	233,297	0.74	-6.33	3.77	0.36	-42.2	5.92	0.33	-39.3	5.99
	Northern Europe	145	229,419	0.67	-9.38	2.77	0.35	-44.93	3.76	0.19	-18.29	4.01
**NO2**	Europe	1,867	4,650,144	0.79	-1.99	8.99	0.51	-4.3	13.72			

Variable	region	Nsite	Nobs	QML			CAMSRA			MERRA-2		
				Cor	NMB (%)	NRMSE (%)	Cor	NMB (%)	NRMSE (%)	Cor	NMB (%)	NRMSE (%)
	Western Europe	1,030	2,454,793	0.81	1.84	8.18	0.53	10.8	12.44			
	Southern Europe	599	1,799,751	0.78	-4.75	10.29	0.47	-21.94	15.07			
	Eastern Europe	177	243,685	0.78	-3.85	11.51	0.5	-29.09	15.35			
	Northern Europe	61	151,915	0.8	7.99	11.57	0.45	0.59	16.17			
O3 8h	Europe	2,021	5,743,369	0.9	0.24	3.20	0.64	-13.81	7.72			
	Western Europe	1,030	3,084,795	0.93	-0.12	3.16	0.69	-17.44	8.98			
	Southern Europe	731	2,225,904	0.88	0.33	4.06	0.57	-7.65	8.28			
	Eastern Europe	189	326,843	0.77	0.23	4.83	0.56	-14.79	2.22			
	Northern Europe	71	105,827	0.83	-0.73	6.04	0.53	-16.08	10.18			

Table S5. Annual Performance of Temporal Cross-Validation in QML Products

Year	PM25			PM10			NO2			O3 8H		
	Nobs	Cor	NRMSE (%)	Nobs	Cor	NRMSE (%)	Nobs	Cor	NRMSE (%)	Nobs	Cor	NRMSE (%)
**2003**	23,194	0.84	2.65	141,927	0.82	3.53	157,924	0.87	8.91	182,529	0.94	4.68
**2004**	45,304	0.79	2.85	124,557	0.82	2.52	173,094	0.88	7.52	202,711	0.93	5.37
**2005**	60,153	0.83	1.99	175,311	0.82	2.12	197,537	0.88	7.93	220,673	0.93	4.75
**2006**	68,158	0.85	2.14	180,594	0.82	2.58	202,591	0.88	7.98	231,113	0.94	4.66
**2007**	85,605	0.85	2.17	211,542	0.81	1.95	200,691	0.88	7.84	226,347	0.94	5.08
**2008**	120,205	0.83	3.61	223,958	0.82	2.12	216,634	0.89	7.5	240,322	0.94	5.31
**2009**	207,265	0.86	1.58	238,979	0.82	2.17	216,543	0.9	7.3	238,871	0.94	5
**2010**	266,339	0.86	1.79	212,193	0.82	2.53	219,231	0.89	7.47	241,350	0.93	4.9
**2011**	304,532	0.88	1.6	266,227	0.85	1.91	220,554	0.89	7.16	239,512	0.93	5.55
**2012**	270,896	0.77	4.48	213,145	0.69	2.83	146,337	0.88	7.51	162,799	0.92	6.43
**2013**	262,770	0.84	1.71	150,843	0.8	1.62	306,578	0.84	7.38	438,092	0.88	4.37
**2014**	289,009	0.86	1.91	151,163	0.82	2.36	365,789	0.86	6.68	486,890	0.88	3.42
**2015**	296,851	0.88	1.42	202,069	0.84	2.5	387,917	0.86	7.06	515,159	0.9	5.21
**2016**	258,390	0.88	2.48	167,603	0.83	1.89	400,716	0.85	7.19	533,326	0.89	4.31
**2017**	313,668	0.88	1.57	210,364	0.82	1.68	408,740	0.87	6.95	521,426	0.9	3.71
**2018**	312,782	0.87	1.86	164,852	0.81	1.65	418,451	0.86	6.28	533,397	0.91	3.79
**2019**	250,186	0.87	1.24	191,598	0.81	1.58	410,817	0.86	6.01	528,852	0.91	4.46
**Total**	3,435,307	0.86	2.05	3,226,925	0.81	2.19	4,650,144	0.87	7.15	5,743,369	0.91	4.55

Table S6. Descriptive statistics of observed and correpsonding QML grid-estimated PM2.5, PM10, NO2, MDA8(maximum daily 8h average) O3 concentrations in different regions (South, East, West, and North) of Europe.

Notes: Average Annual percentages Changes (in %, calculated using Theil-Sen slope dividing mean estimates).

				Observations			Corresponding QML grid-predictions (0.1 degree)			Correlation	
**Variable**	region	Nsite	Nobs	Mean (SD)	Median (IQR)	Annual Change (%)	Mean (SD)	Median (IQR)	Annual Change (%)	Values	Annual Change
**pm25**	Europe	1,310	3,435,307	15.42 (14.45)	12.04 (6.80 , 19.00)	-2.30	14.86 (10.95)	12.44 (7.82 , 18.45)	-2.26	0.85	0.91
	Western Europe	616	1,379,372	16.30 (15.25)	12.91 (7.40 , 20.10)	-2.42	15.75 (11.56)	13.18 (8.34 , 19.73)	-2.20	0.86	0.92
	Southern Europe	500	1,442,244	14.76 (12.76)	12.00 (7.00 , 18.00)	-2.29	14.21 (9.78)	12.12 (7.90 , 17.29)	-2.36	0.84	0.88
	Eastern Europe	85	192,279	18.51 (18.50)	14.32 (8.58 , 22.62)	-2.12	17.59 (13.20)	14.74 (9.63 , 21.86)	-2.12	0.82	0.92
	Northern Europe	109	421,412	8.66 (8.54)	6.16 (4.01 , 11.12)	-2.42	8.32 (5.50)	6.93 (4.46 , 10.74)	-2.20	0.85	0.86
**pm10**	Europe	2,438	3,226,925	27.68 (18.72)	23.27 (16.02 , 34.00)	-2.74	26.93 (14.43)	23.84 (17.55 , 32.56)	-2.61	0.82	0.88
	Western Europe	1,146	1,467,153	26.50 (16.21)	23.00 (16.17 , 32.20)	-2.60	25.69 (12.38)	23.09 (17.37 , 30.98)	-2.49	0.82	0.89
	Southern Europe	849	1,297,056	27.58 (18.00)	23.45 (16.00 , 34.00)	-3.06	27.00 (13.96)	24.17 (17.59 , 33.08)	-3.07	0.82	0.87
	Eastern Europe	298	233,297	38.57 (32.45)	30.20 (19.50 , 46.50)	-2.34	37.20 (24.51)	30.75 (21.55 , 44.93)	-2.19	0.79	0.85
	Northern Europe	145	229,419	21.19 (17.46)	16.90 (11.85 , 25.25)	-2.70	19.40 (9.66)	17.49 (12.79 , 23.93)	-2.40	0.81	0.86
**no2**	Europe	1,867	4,650,144	19.41 (15.55)	15.37 (7.99 , 26.67)	-2.37	18.94 (13.10)	15.99 (9.29 , 25.55)	-2.19	0.87	0.83

				Observations			Corresponding QML grid-predictions (0.1 degree)			Correlation	
**Variable**	region	Nsite	Nobs	Mean (SD)	Median (IQR)	Annual Change (%)	Mean (SD)	Median (IQR)	Annual Change (%)	Values	Annual Change
	Western Europe	1,030	2,454,793	19.46 (14.21)	16.05 (9.00 , 26.41)	-2.26	19.16 (12.22)	16.59 (10.22 , 25.44)	-2.02	0.88	0.83
	Southern Europe	599	1,799,751	19.76 (16.65)	14.92 (7.25 , 27.65)	-2.61	19.16 (13.89)	15.65 (8.58 , 26.29)	-2.49	0.86	0.85
	Eastern Europe	177	243,685	19.29 (19.12)	14.00 (6.18 , 25.70)	-1.13	18.10 (14.79)	14.86 (7.88 , 24.19)	-1.34	0.86	0.78
	Northern Europe	61	151,915	13.82 (15.55)	8.65 (2.44 , 19.20)	-2.74	13.26 (13.21)	9.72 (2.74 , 18.84)	-2.50	0.94	0.79
**o3_8h**	Europe	2,021	5,743,369	70.56 (28.43)	70.03 (51.25 , 88.66)	0.21	70.75 (25.67)	70.62 (53.05 , 87.56)	0.25	0.91	0.90
	Western Europe	1,030	3,084,795	68.07 (28.95)	66.75 (48.31 , 85.83)	-0.07	68.10 (26.59)	67.08 (49.43 , 84.51)	0.05	0.93	0.84
	Southern Europe	731	2,225,904	74.98 (27.95)	75.62 (56.87 , 93.12)	0.58	75.33 (24.76)	76.40 (59.38 , 92.07)	0.71	0.89	0.92
	Eastern Europe	189	326,843	65.91 (27.69)	65.01 (45.86 , 84.25)	-0.24	66.45 (23.04)	66.13 (49.24 , 82.47)	-0.10	0.84	0.86
	Northern Europe	71	105,827	65.61 (18.33)	65.08 (53.09 , 77.75)	-0.18	65.79 (15.17)	65.10 (55.10 , 76.71)	-0.14	0.85	0.89

Table s7. European population proportion (%) exposed to at least 1 day per year of multi-pollutant compound unclean-air days and four major combinations.

Region	Period	All Compound Days	PM25-PM10 Compound Days	PM25-PM10-NO2 Compound Days	PM25-NO2 Compound Days	PM25-O3 Compound Days
**Europe**	2003-2011	96.61	84.45	74.12	70.24	64.52
	2012-2019	86.26	47.94	44.69	59.69	59.85
**Western Europe**	2003-2011	99.36	81.76	90.68	88.51	74.17
	2012-2019	93.80	38.03	56.18	76.76	66.61
**Southern Europe**	2003-2011	96.81	91.02	65.52	69.13	60.02
	2012-2019	76.29	52.68	36.92	53.51	58.11
**Eastern Europe**	2003-2011	99.91	98.76	53.98	35.54	58.31
	2012-2019	94.63	83.45	32.72	34.82	60.05
**Northern Europe**	2003-2011	57.80	41.71	6.44	11.29	4.74
	2012-2019	17.65	7.86	0.00	0.00	0.15

Table s8. Average unclean-air exposure time (unit: days) per year for multi-pollutant compound unclean-air days and four major combinations..

Region	Period	All Compound Days	PM25-PM10 Compound Days	PM25-PM10-NO2 Compound Days	PM25-NO2 Compound Days	PM25-O3 Compound Days
Europe	2003-2011	54.87	15.35	14.27	17.59	4.62
	2012-2019	23.42	4.22	3.61	9.74	5.30
Western Europe	2003-2011	53.58	8.92	13.47	23.99	4.36
	2012-2019	24.32	3.87	3.12	12.80	4.16
Southern Europe	2003-2011	66.11	12.93	22.72	17.28	8.33
	2012-2019	31.02	3.18	8.20	9.55	8.85
Eastern Europe	2003-2011	63.36	40.29	12.86	4.34	3.18
	2012-2019	20.52	7.36	1.74	4.10	6.77
Northern Europe	2003-2011	3.08	1.93	0.25	0.30	0.25
	2012-2019	0.44	0.24	0.03	0.08	0.07

Table s9. Comparison in European population proportion suffering from long-term unclean-air (exceeding WHO annual or peak season guideline) for PM2.5, PM10, NO2 and Ozone among different studies

Note: our study based on grid estimation in 36 European countries; EEA based on urban population with available site observations among EU-27; WHO based on 3654 human settelments with available site observations in 48 countries of European region. For the WHO report, they used the average concentration of 2010-2019 to estimate European population proportion suffering from long-term unclean-air. While calculating the indicator for PM, WHO using PM2.5 as the measure if their settlement have PM2.5 observations, otherwise used PM10 and corresponding guideline instead. Moreover, EEA and WHO did not give the peak season compliance for Ozone, so this table does not include them. As almost no estimated grid cell is found to meet the ozone peak season guideline, here we used interim target 2 (>70 ug/m3) of Ozone instead.

Year	PM2.5		PM10		PM	NO2			Ozone
	This study	EEA	This study	EEA	WHO	This study	EEA	WHO	This study
2003	99.95%		97.74%	99.00%		97.11%	100.00%		99.08%
2004	99.96%		95.58%	97.00%		95.24%	100.00%		89.41%
2005	99.96%		95.61%	98.00%		95.92%	99.00%		88.54%
2006	99.97%	100.00%	96.86%	99.00%		95.77%	99.00%		98.99%
2007	99.93%	100.00%	96.51%	99.00%		95.29%	99.00%		89.43%
2008	99.87%	99.00%	94.14%	97.00%		94.18%	98.00%		93.17%
2009	99.81%	99.00%	94.12%	96.00%		93.75%	98.00%		90.06%
2010	99.85%	100.00%	93.48%	95.00%	86%	93.63%	99.00%	77%	93.33%
2011	99.82%	100.00%	94.62%	95.00%		93.72%	97.00%		93.78%
2012	99.71%	98.00%	83.76%	95.00%		92.81%	98.00%		89.22%
2013	99.52%	97.00%	84.15%	94.00%		91.66%	98.00%		96.77%
2014	99.58%	100.00%	81.07%	94.00%		90.30%	97.00%		94.03%
2015	98.19%	97.00%	87.65%	94.00%		90.66%	97.00%		95.09%
2016	97.89%	97.00%	81.64%	88.00%		92.01%	97.00%		89.48%
2017	97.71%	97.00%	82.60%	89.00%		89.36%	97.00%		94.59%
2018	98.16%	98.00%	85.81%	91.00%		89.45%	97.00%		99.01%
2019	98.10%	97.00%	80.15%	81.00%		86.34%	94.00%		92.66%

Supplementary Reference

1. Zhao-Yue Chen *et al. A Pan-European, Quantile Machine Learning (QML) Based, Total, Fine-Mode and Coarse-Mode Aerosol Depth Dataset (QML AOD))*. https://doi.org/10.5281/zenodo.8315721 (2023)
2. Meijer, J. R., Huijbregts, M. A. J., Schotten, K. C. G. J. & Schipper, A. M. Global patterns of current and future road infrastructure. *Environmental Research Letters* **13**, (2018).
3. Ching, J. *et al.* WUDAPT: An urban weather, climate, and environmental modeling infrastructure for the anthropocene. *Bull Am Meteorol Soc* **99**, 1907–1924 (2018).
4. Kummu, M., Taka, M. & Guillaume, J. H. A. Gridded global datasets for gross domestic product and Human Development Index over 1990–2015. *Sci Data* **5**, 1–15 (2018).
